# Cocoon-Spinning Behavior and 20-Hydroxyecdysone Regulation of Fibroin Genes in *Plutella xylostella*

**DOI:** 10.3389/fphys.2020.574800

**Published:** 2020-12-15

**Authors:** Yan Shi, Gan-Lin Lin, Xiu-Lian Fu, Mike Keller, Guy Smagghe, Tong-Xian Liu

**Affiliations:** ^1^Key Lab of Integrated Crop Pest Management of Shandong Province, College of Plant Health and Medicine, Qingdao Agricultural University, Qingdao, China; ^2^School of Agriculture, Food and Wine, University of Adelaide, Glen Osmond, SA, Australia; ^3^Department of Plants and Crops, Faculty of Bioscience Engineering, Ghent University, Ghent, Belgium

**Keywords:** *Plutella xylostella*, diamondback moth, cocoon-spinning behavior, 20E, fibroin genes

## Abstract

The diamondback moth *Plutella xylostella* is a serious pest of crucifers. It has high reproductive potential and is resistant to many insecticides. Typically, the last-instar larvae of *P. xylostella*, before pupation, move to the lower or outer plant leaves to make a loose silk cocoon and pupate inside for adult formation. To better understand this pivotal stage we studied the cocoon-spinning behavior of *P. xylostella* and measured three successive phases by video-recording, namely the selection of a pupation site, spinning a loose cocoon and padding the scaffold cocoon. Subsequently, we cloned three fibroin genes related to cocoon production, i.e., fibroin light chain (Fib-L), fibroin heavy chain (Fib-H), and glycoprotein P25. A spatio-temporal study of these three fibroin genes confirmed a high expression in the silk glands during the final larval instar silk-producing stage. In parallel, we did an exogenous treatment of the insect molting hormone 20-hydroxyecdysone (20E), and this suppressed fibroin gene expression, reduced the normal time needed for cocoon spinning, and we also observed a looser cocoon structure under the scanning electron microscope. Hence, we demonstrated that the expression levels of key genes related to the synthesis of 20E [the three Halloween genes *Spook (Spo), Shadow* (*Sad)*, and *Shade* (*Shd*)] decreased significantly during spinning, the expression of the 20E receptor (*EcR* and *USP*) was significantly lower during spinning than before spinning, and that the expression levels of *CYP18-A1* related to 20E degradation were significantly up-regulated during spinning. The significance of the cocoon and the effects of 20E on the cocoon-spinning behavior of *P. xylostella* are discussed.

## Introduction

Cocoon spinning behaviors have been described in many insect species before like, the domesticated silkworm *Bombyx mori* (Yokoyama, 1951; Kiyosawa et al., 1999; Chen et al., 2012a,b; Guo et al., 2016), the giant silkworm *Hyalophora cecropia* (Van der Kloot and Williams, 1953), and the Chinese oak silkworm *Antheraea pernyi* (Lounibos, 1975, 1976). These authors described the major features of the stereotyped spinning movements and the effects of environmental conditions on the morphology, structure and mechanical properties. Yagi (1926) classified cocoons into four types by their formation of the exit hole of the adults and the modes of attachment. They were: (1) stalked and closed, (2) stalkless and closed, (3) stalkless and open, and (4) stalked and open. The cocoon of *P. xylostella* belongs to the third type.

The fibrous silk core is generally comprised of a three-protein complex, including fibroin light chain (Fib-L), fibroin heavy chain (Fib-H) and glycoprotein P25 (Sehnal and Zurovec, 2004; Sutherland et al., 2010). The development and function of the silk gland are under hormone control (Chaitanya and Aparna, 2010; Boulet-Audet et al., 2016). Ecdysone is the fundamental steroid hormone, released from the prothoracic glands in insects. The dormant state of ecdysone is discharged into the hemolymph and converted into 20-hydroxyecdysone (20E) in the peripheral tissues (Li et al., 2019). Most hormonal studies on *Fib-H, Fib-L* and *P25* are limited to *B. mori, Corcyra cephalonica* and *Sylepta derogate* (Miao et al., 2004; Chaitanya and Aparna, 2010; Su et al., 2015). In *B. mori*, the ecdysteroids are essential for appropriate function of silk gland. In *C. cephalonica*, studies revealed that 20E modulates the expression of the *Fib-L, Fib-H*, and *P25* genes at the mRNA level. These reports indicated that larval development and silk gland function in these insects are regulated by 20E. Although the synthesis and secretion of silk fibroin have been studied in detail in model insects like *B. mori*, the cocoon behavior, these silk genes and the hormonal regulation have not been uncleared in the diamondback moth, which is an agricultural pest that causes high damage to cruciferous vegetables. It has a high reproductive potential, a wide distribution in the world, and is resistant to many insecticides.

In this study, we therefore studied the cocoon-spinning behavior of *P. xylostella* larvae by video-recording, and subsequently we examined the silk genes *Fib-L, Fib-H*, and *P25* by cloning and a spatio-temporal molecular characterization, and tested the effect of an exogenous treatment of 20E on their expression and the cocoon formation under the scanning electron microscope (SEM). Finally, we measured the expression of three 20E-related genes for synthesis [namely the Halloween genes *Spook* (*Spo*)*, Shadow* (*Sad*), and *Shade* (*Shd*)], its receptor (the ecdysone receptor *EcR* and Ultraspiracle *USP*) and degradation (*CYP18-A1*) at the moments of before, during and after the spinning behavior. We selected *Spo* (Cyp307a1) and *Sad* (Cyp315a1) encoding cytochrome P450 enzyme needed for 20E biosynthesis (Namiki et al., 2005; Gilbert, 2008). *Shd* (Cyp314a1) is the final step in the biosynthetic pathway for an ecdysone 20-monooxygenase enzyme responsible (Petryk et al., 2003). On EcR and USP, they form the functional nuclear heterodimer receptor for 20E, which modulates insect molting and metamorphosis (reviewed in Fahrbach et al., 2012). Finally, CYP18-A1 encodes a P450 enzyme ecdysteroid 26-hydroxylase that is a major 20E hormone inactivation enzyme (Guittard et al., 2011; Li et al., 2014). We believe these data should allow to better understand the significance of the cocoon, the effects of 20E on cocoon spinning behavior of *P. xylostella*, and the possible use of cocoon manipulation for pest management.

## Materials and Methods

### Insect Rearing and Cocoon Collection

The *P. xylostella* used for this works were collected from a cabbage (*Brassica rapa* L. ssp. *pekinensis*) field in Jiaozhou, Qingdao (36°16′39″N, 120°00′41″E) in 2018, and has since been reared in laboratory at the Qingdao Agriculture University. *Plutella xylostella* were rearing in a 16:8 h (L: D) photoperiod maintained insectary cages at 20–25°C and 50–70% relative humidity (RH). Adults were fed 10% honey solution, and larvae were fed with fresh Chinese cabbage (*Brassica rapa* L. ssp. *pekinensis*) leaves until they started to spin cocoons. For collecting cocoon, we used dissecting scissors to gently peel off the pupa from the cocoon to observe using electron microscope.

### Observation of Cocoon-Spinning Behavior

The cocoon-spinning behavior of *P. xylostella* larvae was recorded using a Canon video camera (HFR86, Canon, Tokyo, Japan) and analyzed by playing the video recordings back on a computer. Each larval cocoon-spinning behavior was recorded from the time when the larva started selection of its pupation site and scaffolding until the completion of the inner cocoon. For the observation and recording of cocoon, we totally recoded 15 larvae cocooning.

### Sequence Alignment and Phylogenetic Analysis

DNAMAN 6.0 software was used to predict the open reading frame (ORF). NCBI CDD database was applied to identify the conserved domains. The amino acid sequences of homologous Halloween genes and ecdysone receptor genes were aligned with Clustal W2.0. Phylogenetic tree was constructed using the method of neighbor-joining (NJ) with a bootstrap value of 1000 replicates.

### Cloning and Real-Time Quantitative PCR (RT-qPCR)

Using the genome database and transcriptome of *P. xylostella* (Tang et al., 2014) and identified gene sequence in *B. mori*, the partial gene of fibroin-H and the open reading frame of the fibroin-L, P25, the three 20E-biosynthesis Halloween genes *Spo* (Cyp307a1), *Sad* (Cyp315a1), and *Shd* (Cyp314a1), and the ecdysone receptor (*EcR*), Ultraspiracle (*USP*) and the 20E-catabolizing *CYP18-A1* genes were identified by performing a homologous search. RNA was extracted from the final instar larvae using Trizol reagent (Promega, Beijing, China). Single-strand cDNA was prepared using the PrimeScript first-strand synthesis system (Promega, Beijing, China). Primers were designed based on the genome data of *P. xylostella* (Supplementary Table 1). Using high-fidelity DNA polymerase PrimeSTAR (Takara, Dalian, Liaoning, China), the partial of *fib-H* and the open reading frame of *fib-L* and *P25* were amplified. We refer to Shi et al. (2017) for the setting of the amplified PCR program. Subsequently, PCR products were purified and cloned into vector and sequenced (TsingKe, Qingdao, China). Total RNA of four replicates was obtained for the 20E treatment and the control. Single-strand cDNA was prepared using the PrimeScript first-strand synthesis system (Takara). We performed the RT-qPCR with the protocol as described by Shi et al. (2017), and *Actin* and *rpl32* were used as reference genes (Sun et al., 2013). Four biological replicates and each replicate including 3 individuals were performed for each developmental stage (egg, larva, pupa, and adult), larval tissue (midgut, central nervous system, silk gland, cuticle, and Malpighian tubules), before and after spinning, and the data were analyzed with the 2^−ΔΔCt^ method (Pfaffl, 2001).

### Effect of 20E on the Expression of Fibroin-H, Fibroin-L, and P25

A 5 μg/μL solution of 20E (Sigma-Aldrich, St. Louis, MO, USA) was dissolved in 95% ethanol. Each 3th instar larva in the 20E-treatment group was injected with a dose of 500 ng 20E solution by micro 4 injector (World precision instruments). Each larva in the control group was injected with an equal volume of ethanol. Four injected larvae were randomly taken at 3, 6, and 12 h after injection, and the RNA of the silk gland (four biological replicates per treatment and each replicate included 20 silk glands) was separated to check the relative expression levels using qPCR, as described above.

### SEM Observation of Cocoon

A cocoon was removed from the leaf surface, pasted on superconducting tape and sputter coated, and a hole (3.5 mm in diameter) was punched in each cocoon with a steel punching device. Then, the fibers were put into fine tubes and crushed perpendicular to the fiber lengths. The tubes with fibers were pasted on tape and sputter coated. Then the coated samples were observed in a SEM (Jeol Neoscope JCM-5000, Nikon, Tokyo, Japan) at 15 kV voltage. Both the 20E-treated cocoons and the control cocoons were observed with four replications each.

### Data Analysis

To analyze the significance of time spent in each steps, the gene expression profile in different tissues and the relative transcripts of key genes related to 20E in three steps of spinning, we used the one-way ANOVA followed by a Tukey's honest significant difference multiple comparison test (*P* < 0.05). Other data in the treatment and control groups with four replications were analyzed using a Student's *t*-test (^*^P < 0.05) to determine the significant differences by SPSS 20.0 (SPSS, Chicago, IL, USA), and data are presented as means ± SD.

## Results

**Figure 1:**
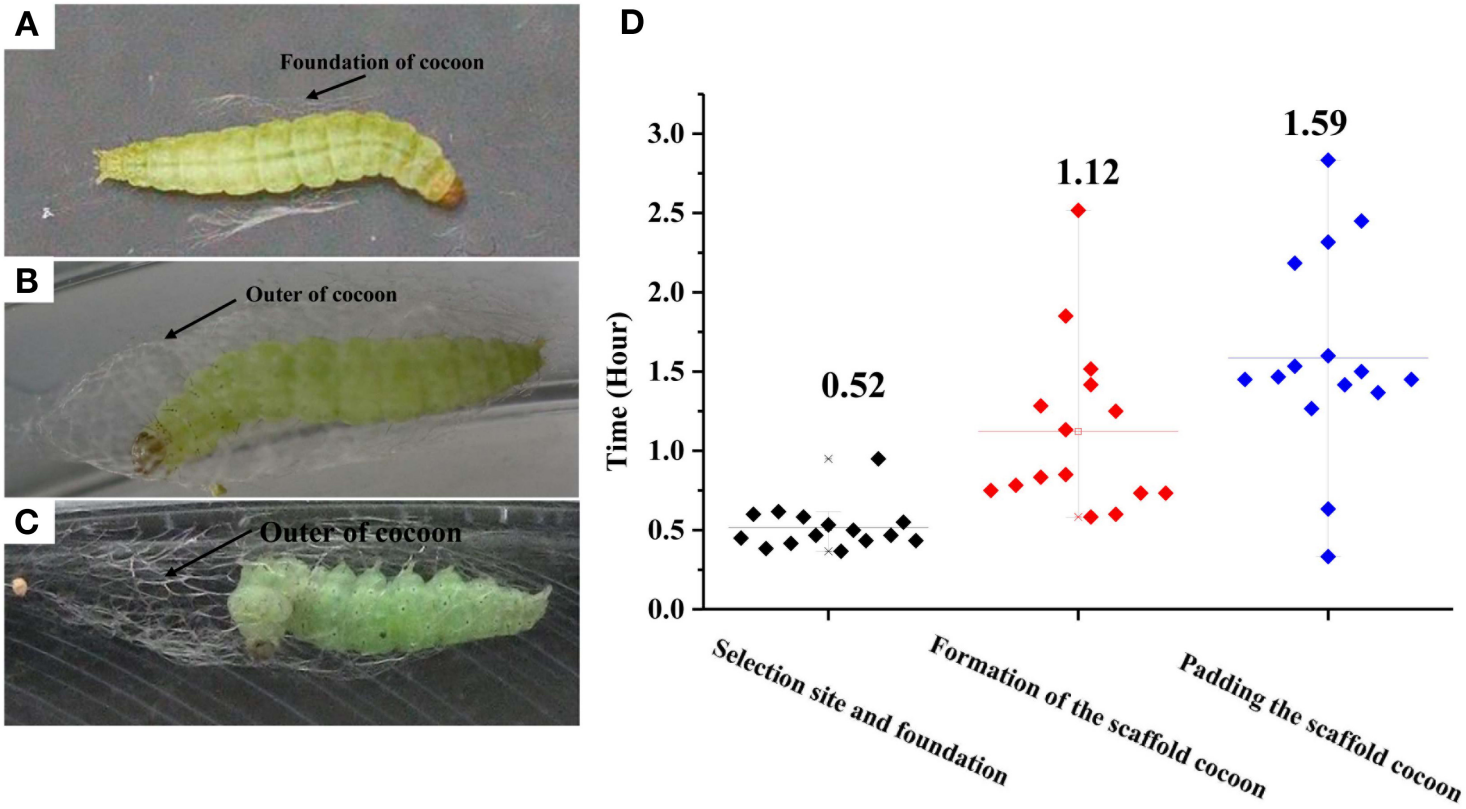
Photographs of the three successive phases for cocoon construction by *Plutella xylostella* larvae. **(A)** Selecting a pupation site and building a foundation. **(B)** Formation of the scaffold cocoon; **(C)** Padding the scaffold cocoon; and durations **(D)**. Different letters indicate statistical differences. The left y-axis indicates the time spent in each phases of cocooning; the x-axis shows three phases of cocooning.

The construction of a cocoon by a final instar larva of *P. xylostella* can be divided into three phases separable by their movement patterns: (1) selection of a pupation site and construction of a cocoon foundation, (2) formation of a scaffold cocoon, and (3) internal padding the scaffold cocoon. Before the *P. xylostella* larva begins cocoon construction it evacuates its gut, wanders for a period of time, and then precedes to pupate. It first makes a foundation for the cocoon by spinning a small amount of silk on the leaf surface. By strengthening interconnecting strands with applications of silk, the larva produces a fibrous network which forms the foundation for the incipient cocoon. The larva remains attached in this location until the spinning process is completed. Under laboratory conditions, each larva spent an average of 0.52 h in this initial phase of cocoon construction. More than half of the cocoon construction time was used to create the foundation (Figures 1A,D). The mature larva first spins a wide foundation of silk, and then gradually concentrates its spinning area to a size that is similar to the final cocoon. The larva spins a thin outer cocoon layer after completion of the foundation. Once the larva begins spinning, it continues without stopping and this process lasts an average of 3.23 h. During this time, it repeats the fixation and movement of the posterior half of the larval body with abdominal and caudal legs. The larva fixes the posterior half of the body, and spins by moving the anterior half of the body. The larva spends an average of 1.12 h for the construction of the outer cocoon (Figures 1B,D, Supplementary Video 1). In the final spinning cycle, the larva frequently interrupts its extension-recovery sequence and remains motionless. The intervals between the motionless periods gradually use greater proportions of successive, head-up cycles until the spinning behavior ends. At the time, the larva remains still in the head-up position. Padding the scaffold cocoon requires a longer time than the first two phases (*t* = 11.14; df = 1, 441; *P* < 0.01; Figures 1C,D).

### Cloning Genes and Expression Profiling

**Figure 2:**
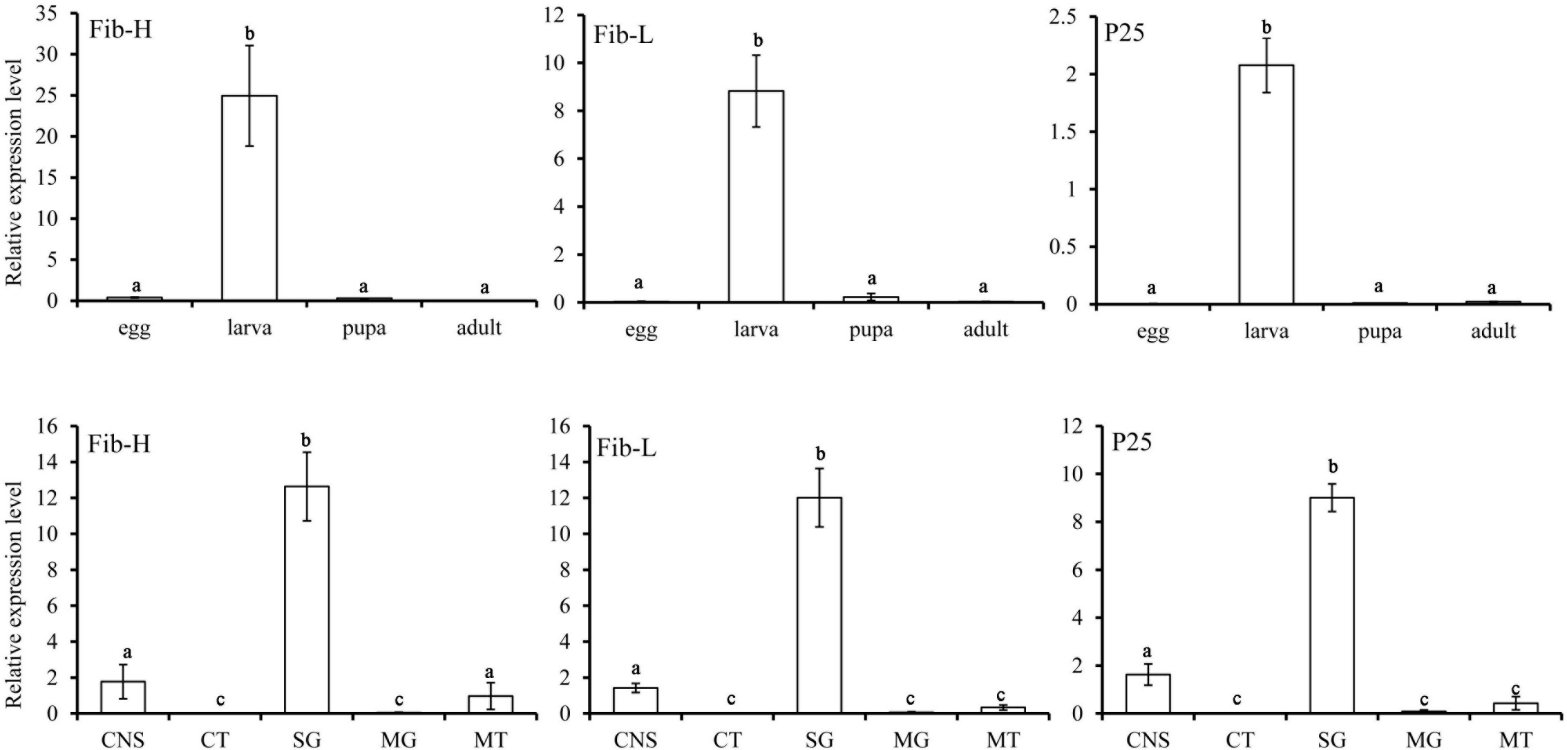
Relative transcripts of *Fib-L, P25*, and *Fib-H* detected by qPCR indifferent developmental stages (egg, larva, pupa, and adult) and different larval tissues of *Plutella xylostella*. Abbreviations used on X-axis: MG, midgut; CNS, central nervous system including brain and ventral nerve cord; SG, silk gland; CT, cuticle; MT, Malpighian tubules.

*P. xylostella Fib-L, P25*, and the 5′end of *Fib-H, EcR, USP*, and *CYP18-A1* consisted of 252, 221, 106, 546, 415, and 532 amino acids, respectively (Supplementary Figure 2). These nucleotide sequences of *P. xylostella* were expressed as in GenBank, respectively (Supplementary Table 1). *Fib-L, P25*, and *Fib-H* genes are regulated in specific developmental stages and in specific tissues (Figure 2). Expression of the *Fib-L, P25*, and *Fib-H* genes occurs mainly in the final instar larvae but not in pupae. The maximum expression of *Fib-L, P25*, and *Fib-H* genes was observed in the silk gland tissue but none is expressed in other tissues (Figure 2). On *EcR, USP*, and *CYP18-A1* of *P. xylostella*, they were cloned and the phylogenetic analysis confirmed that *PxEcR, PxUSP*, and *PxCYP18-A1* are closely related to *EcR, USP*, and *CYP18-A1* from other lepidopteran insects, such as *B. mori, Manduca sexta, Spodoptera exigua* (Supplementary Figure 1).

### Effect of 20E on Fibroin mRNA Expression and Cocoon Spinning Behavior

**Figure 3:**
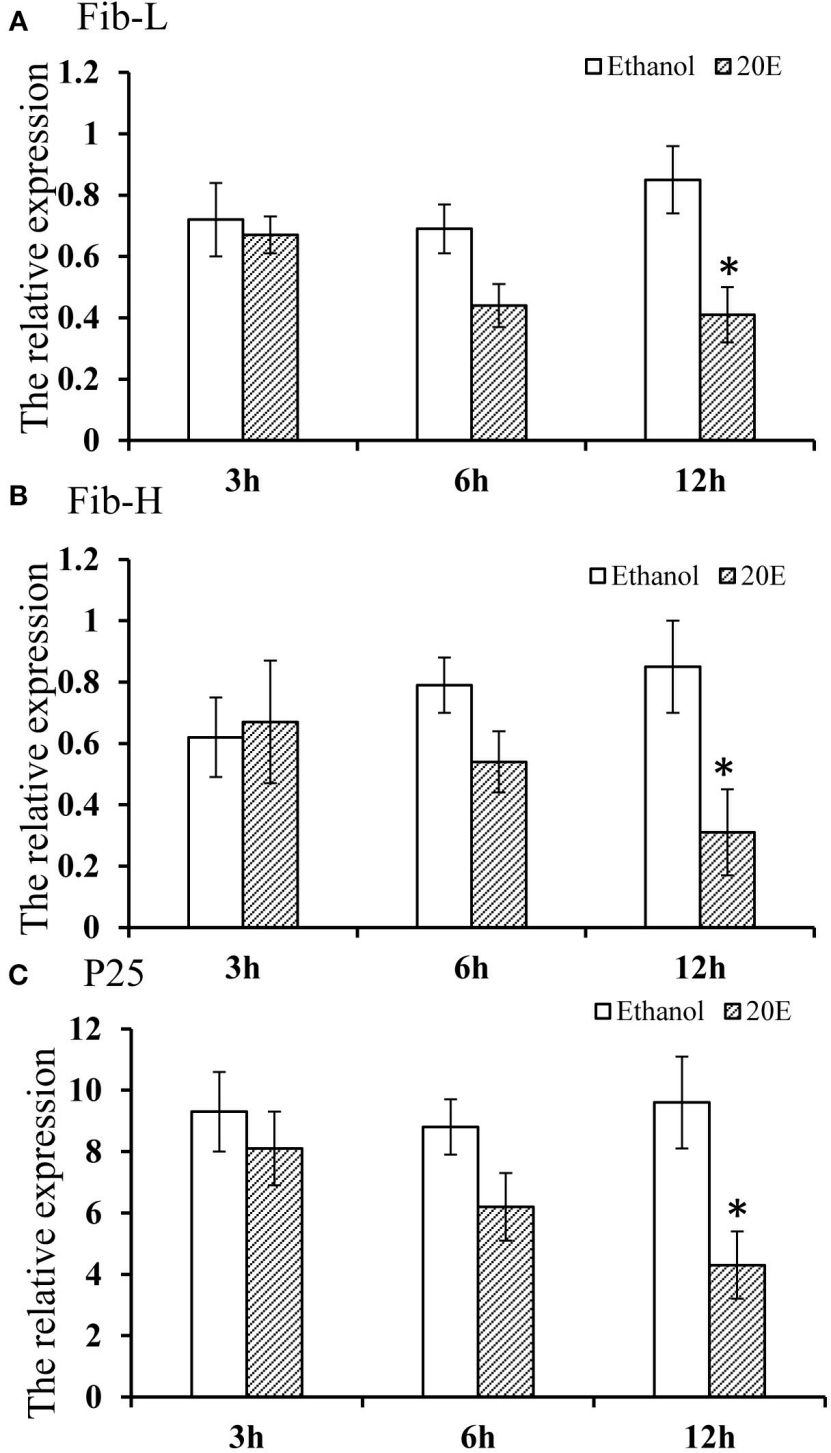
**(A–C)** Effect of 20E on *Fib-L, P25*, and *Fib-H* expression of larval silk glands of *Plutella xylostella* larvae at 3, 6, and 12 h time points in the control (ethanol treated) and 20E-treated groups. Data are means SD. Significant differences were checked using Students *t*-test (*P < 0.05).

**Figure 4:**
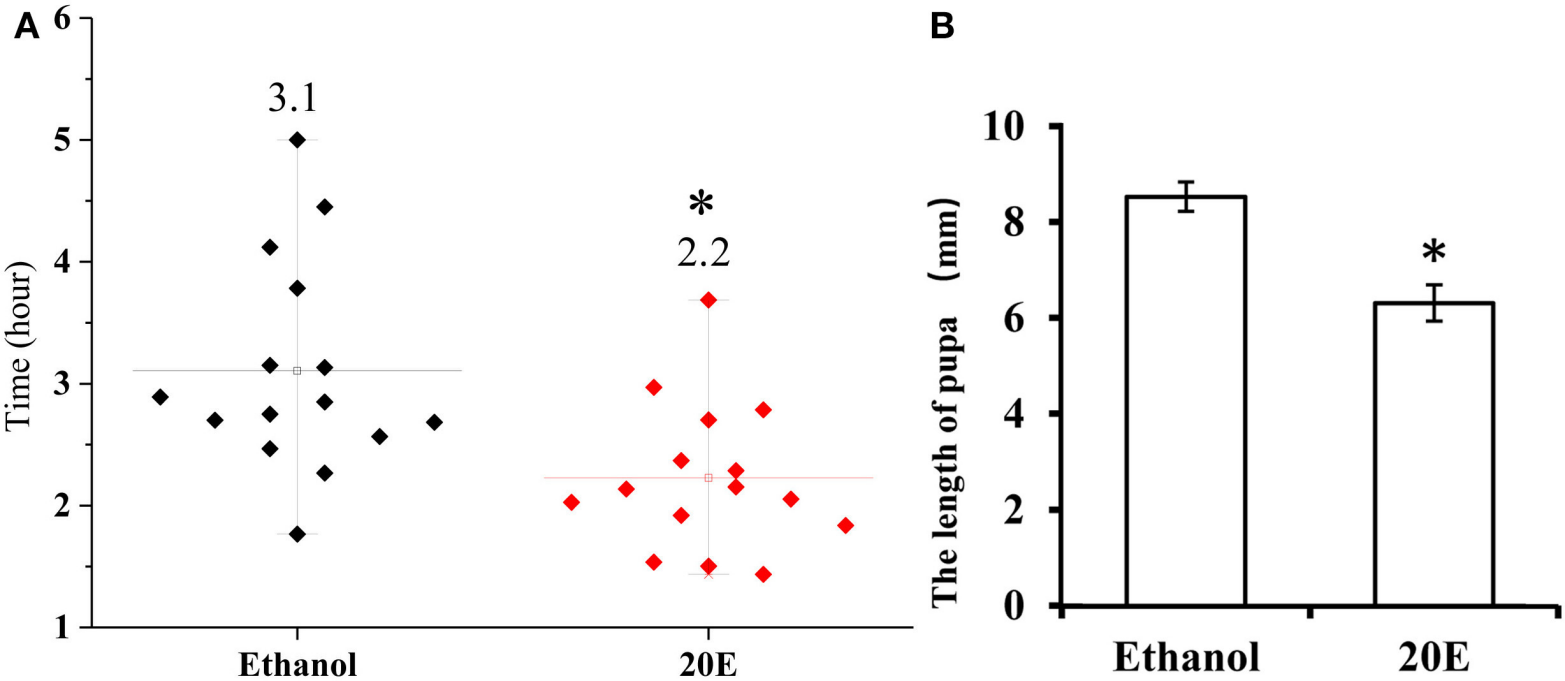
**(A)** Cocoon-spinning times *Plutella xylostella* larvae in the control and the 20E-treated groups. **(B)** The pupa sizes in the control and the 20E-treatedgroups. Data are expressed as means SD. Significant differences were calculated using Students *t*-test (*P < 0.05). The left y-axis indicates the time spent of cocooning; the x-axis shows control and the 20E treated groups.

**Figure 5:**
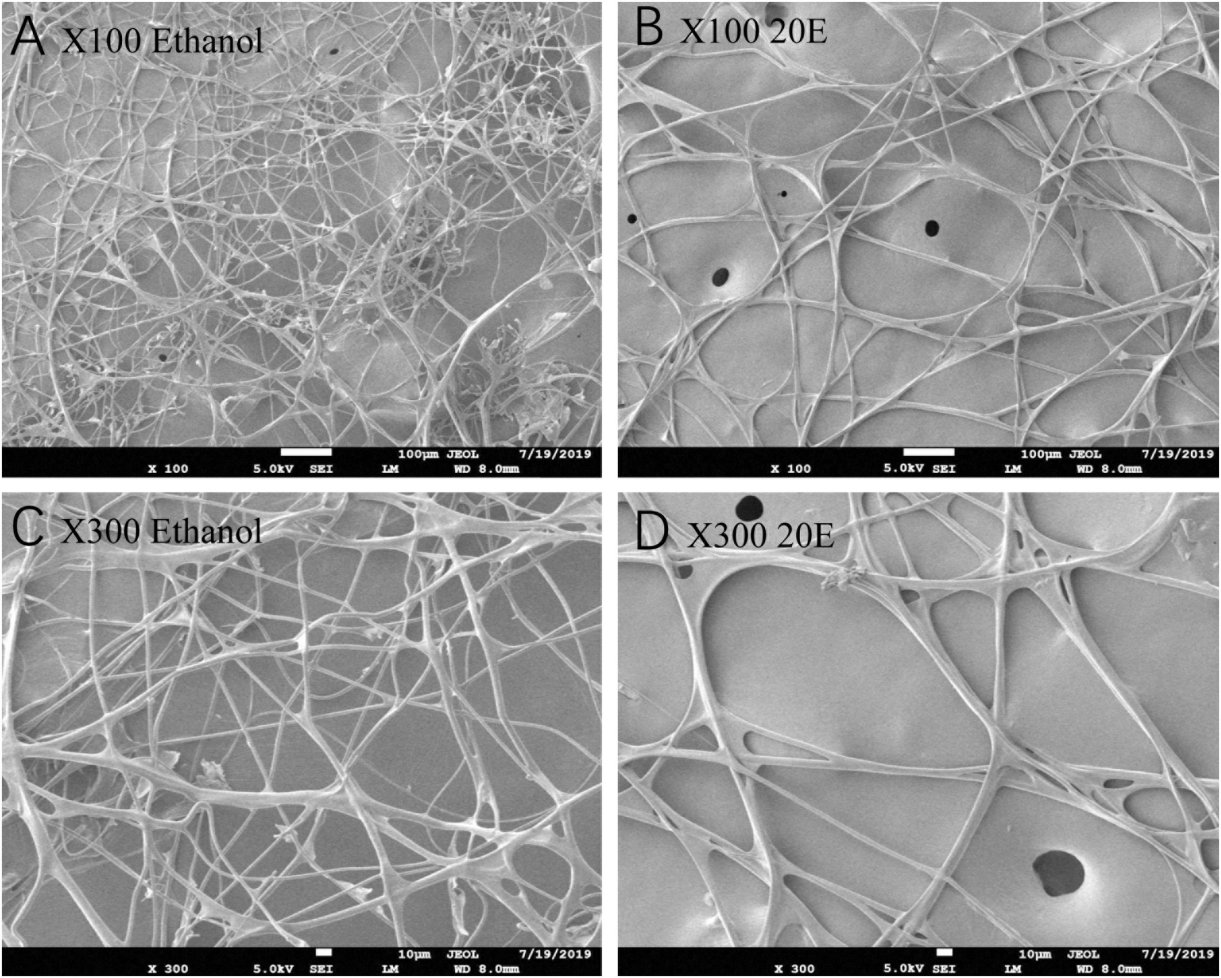
Scanning electron microscopy micrographs of the cocoon looseness of *Plutella xylostella* in the control **(A,C)** and the 20E-treated groups **(B,D)** with 100 and 300 magnification.

The effects of 20E and ethanol on the expression of *Fib-L, Fib-H*, and *P25* genes in silk glands are shown in Figures 3A–C. The transcript levels at the same time points (3, 6, and 12 h after treatment) and the expression levels of *Fib-L, Fib-H*, and *P25* genes were unaltered at 3 and 6 h after treatment. At 12 h after treatment, there was a decline in the expression levels of *Fib-L, Fib-H*, and *P25* genes in the 20E treatment compared with the control (Figures 3A–C). Hence, the 20E-treated group had significantly less spinning time than the control (*t* = 13.11; df = 1, 44; *P* < 0.01; Figure 4A), and also the 20E-treated larvae spun cocoons that were looser and the resulting pupae were smaller than the controls (*t* = 11.54; df = 1, 40; *P* < 0.01; Figure 4B). At same times, the 20E-treatment group showed that the cocoon became thinner leaving only some scaffold silks, while some of the filled silk disappeared (Figure 5).

**Figure 6:**
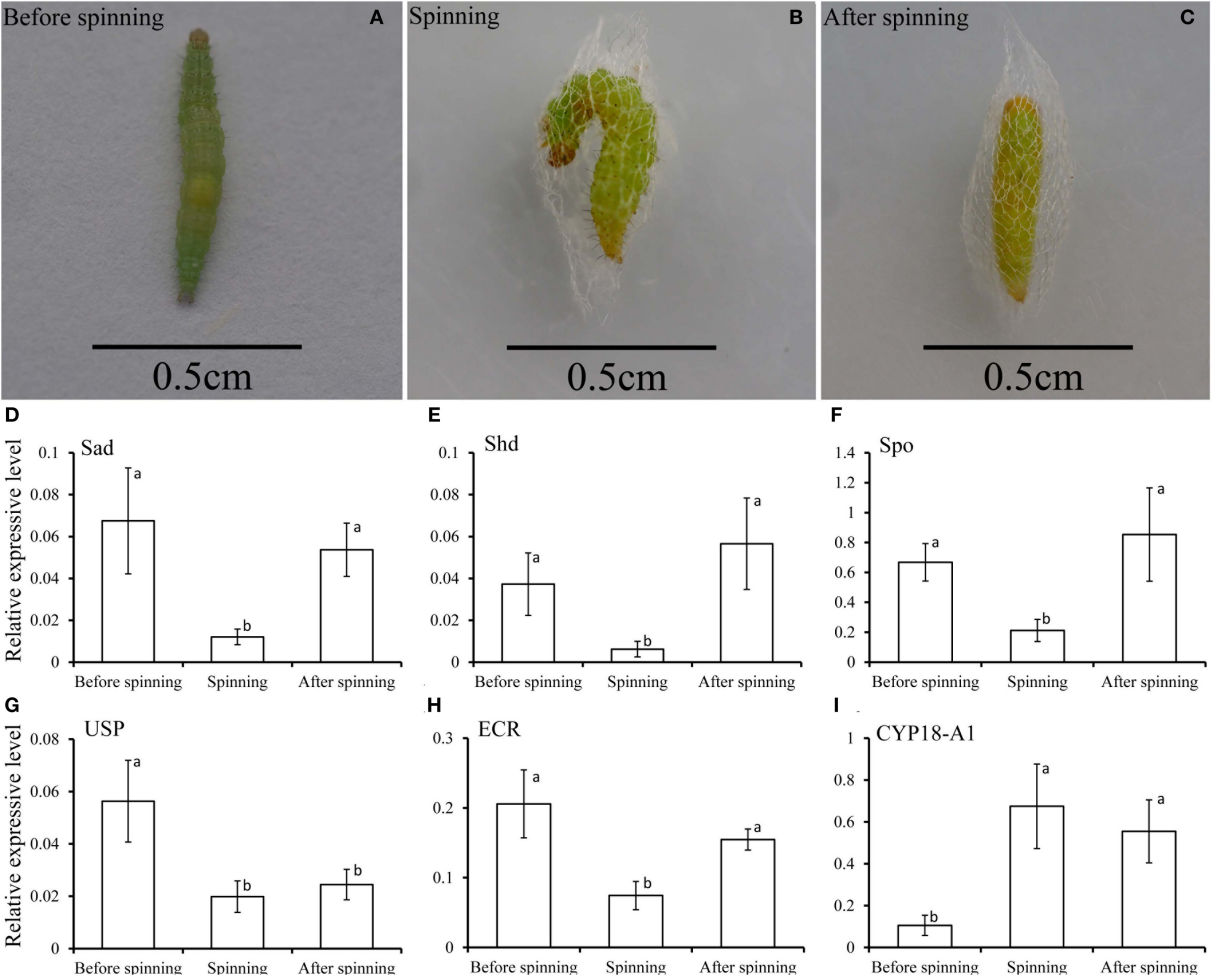
**(A–C)** Respectively represent the before spinning, spinning, and after spinning of the diamondback moth larvae. **(D–I)** Relative transcripts of the key genes related to 20E, including 20E-biosynthesis (*Spo, Sad*, and *Shd*), signaling (*EcR* and *USP*), and degradation *(CYP18-A1)*, detected by qPCR at before, during and after spinning of *Plutella xylostella* mature larvae.

In order to better understand the relationship between the spinning behavior and the 20E treatment (Figures 6A–C), we measured the expression levels of three key genes in the biosynthesis of 20E (the Halloween genes *Spo, Sad*, and *Shd*), its signaling pathway (*EcR* and *USP*) and the degradation of 20E (*CYP18-A1*) in the three periods of before, during and after spinning. The results showed that the expression level of three key genes related to 20E synthesis (*Spo, Sad*, and *Shd*) decreased significantly from high before spinning to low during spinning (Figures 6D–F). For the 20E receptor (*EcR* and *USP*), the expression level also decreased between before and during spinning (Figures 6G,H). In contrast, the expression of the key gene *CYP18-A1* related to degrading 20E, its expression levels were significantly higher during spinning and after spinning as compared to before spinning (Figure 6I).

## Discussion

Cocoons are a pivotal stage in the survival and reproduction of many arthropods. Most researches have focused on the cocoon silk of silkworms and the dragline silk of spiders (Offord et al., 2016; Xu et al., 2018). However, many insect species produce cocoons using a wide variety of spinning behaviors. The spinning behavior of many insects has been described (Lounibos, 1975; Giebultowicz et al., 1980; Stuart and Hunter, 1995; Kiyosawa et al., 1999). The cocoon-spinning behavior of *P. xylostella* consists of three phases. The first phase begins with gut purging behavior. This is distinct, physiologically important and similar to the behavior of *B. mori* (Lounibos, 1975; Stuart and Hunter, 1995; Kiyosawa et al., 1999).

Silk cocoons are composed of fiber proteins (fibroins) and adhesive glue proteins (sericins), which provide a physical barrier that protects the pupa (Chen et al., 2012a,b, 2013). The cocoon silk fibroins consist of a large protein, named as the heavy chain fibroin (Fib-H), and two smaller proteins, named the light chain fibroin (Fib-L) and P25 (Chen et al., 2012b). Insects characterized three forms of silk fibroin structures. These are the Fib-H, Fib-L, and P25 in *B. mori* and *C. cephalonica* (Shimizu et al., 2007; Chaitanya and Aparna, 2010). Our study confirmed the presence of *Fib-L, P25*, and *Fib-H* in *P. xylostella*. We also cloned the *Fib-L, P25*, and a partial *Fib-H* cDNA from *P. xylostella*. Tissue-specific and developmental stage expression of *Fib-L, P25*, and *Fib-H* genes revealed that they are highly expressed in the silk gland only and this during the final larval stage (Figure 2). This indicates that the transcription of *Fib-L, P25*, and *Fib-H* genes is developmental and tissue-specific regulated (Figure 2). These results in *P. xylostella* are consistent with studies in *B. mori, S. derogate*, and *C. cephalonica* (Sehnal and Zurovec, 2004; Chaitanya and Aparna, 2010; Su et al., 2015). Molting occurs mainly in the larval stage, and 20E is the main hormone that regulates the molting process (Li et al., 2019). Based on our current findings and previous studies, the larval stage of *P. xylostella* was selected to study the effects of an exogenous treatment of 20E on these genes and the cocoon behavior and structure. We measured the effect of 20E on the expression of these genes in the larval silk glands. Ecdysteroids cause silk gland degeneration during the larva-pupa molt of *B. mori* (Shimizu et al., 2007). The larva-pupa molt is associated with ecdysteroids. The developmental pattern of *Fib-L, P25*, and *Fib-H* genes in *P. xylostella* showed that their expression dropped when 20E was used to treat the mature larva (Figure 3). Therefore, we speculated that 20E may regulate the spinning behavior of *P. xylostella*. Further, more evidence was obtained when we measured the expression levels of key genes related to 20E, including its biosynthesis, signaling and degradation, in three periods of spinning, namely before, during and after spinning (Figures 6A–C). The results showed that they changed significantly in the three different periods of spinning (Figures 6D–I). Specifically, the three 20E-biosynthesis Halloween genes *Spo, Sad*, and *Shd* decreased from high to low with the appearance of the spinning behavior (Figures 6D–F). Based on the results of Niwa and Niwa (2014), Niwa and Niwa (2016), and Peng et al. (2019) we believe that the expression profile of *Spo, Sad* and *Shd* corresponds with the 20E hormone titer, where an increase in expression corresponds to 20E biosynthesis and a hormone peak rise (Iga and Smagghe, 2010). Hence, Peng et al. (2019) reported on the role of *Shd* in *P. xylostella* where RNAi of Shd significantly reduced the 20E titer and resulted in a longer developmental duration and lower pupation of *P. xylostella* fourth-instar larvae. For *EcR* and *USP*, forming the nuclear ecdysone receptor heterodimer, their expression profile followed that of the 3 Halloween genes, where there was a strong drop from before to during the spinning (Figures 6G,H). This is according to our expectations as before it has been reported by different authors in multiple insects that the presence hormone receptor follows its hormone titer (reviewed in Fahrbach et al., 2012). In contrast, the expression of the 20E-degrading 26-hydroxylase enzyme CYP18-A1, which follows after a peak of 20E to reduce the hormone titer, was strongly increased with the appearance of the spinning behavior. Before it has been reported that 20E hormone was degraded and cleared out of the insect body by CYP18-A1 enzyme and this is required for successful development, specifically post-apolysial processes as ecdysial behavior (Guittard et al., 2011; Li et al., 2014). Indeed this event is consistent with our expectation that the expression level of CYP18-A1 was significantly increased with the spinning behavior of *P. xylostella* (Figure 6I). All these research data demonstrated that the presence of 20E inhibits the cocoon spinning of diamondback moth. Therefore, the treatment of the final instar larvae with exogenous 20E significantly downregulated the expression of these genes in the silk gland and led to the construction of looser cocoons as we saw in the SEM (Figure 5). We also found that 20E reduced the spinning time (Figure 4) and this may be related to the acceleration of pupation. Hence, we note here that a series of studies has reported that 20E and ecdysteroid-mimicking compounds, which can be used as insect growth-regulatory insecticides to control pest insects (irac-online-org), can accelerate larval metamorphosis (Smagghe and Degheele, 1994; Smagghe et al., 2013; Scieuzo et al., 2018; Lin et al., 2019). Sometimes pupation occurs before the larva is mature, resulting in a smaller pupa than normal.

In summary, we described the entire cocoon-spinning process by video-recording and the exact steps and movement, as well as the duration of the process. We also found 20E affected the cocoon-spinning and the structure of the cocoon through modulation of *Fib-L, P25*, and *Fib-H* at the mRNA level in the final instar *P. xylostella* larvae. More studies on cocoon silk-producing insect pests, the function of the cocoon, and the possible use of cocoon manipulation for pest management are needed.

## Data Availability Statement

The raw data supporting the conclusions of this article will be made available by the authors, without undue reservation.

## Author Contributions

YS, GS, and T-XL designed research. YS performed all of the experiments with the help of G-LL and X-LF provided the materials. YS, G-LL, X-LF, and MK analyzed data. YS, MK, GS, and T-XL wrote the paper. All authors contributed to the article and approved the submitted version.

## Conflict of Interest

The authors declare that the research was conducted in the absence of any commercial or financial relationships that could be construed as a potential conflict of interest.

## Supplementary Material

Supplementary Figure 1 Phylogenetic analysis of ECR, USP, and CYP18-A1 homologs from different insect species based on amino acid sequences.

Supplementary Figure 2 Multiple alignments of Fib-L, P25, and a partial of Fib-H in *Bombyx mori, Corcyra cephalonica*, and *Plutella xylostella*.

Supplementary Video 1 The video of diamondback moth larvae spinning and cocooning.

Supplementary Table 1 Primer sequences used in this study.

## References

[B1] Boulet-Audet M. Holland C. Gheysens T. Vollrath F. (2016). Dry-spun silk produces native-like fibroin solutions. Biomacromolecules 17, 3198–3204. 10.1021/acs.biomac.6b00887 27526078 PMC5059755

[B2] Chaitanya R. K. Aparna D. G. (2010). Light chain fibroin and P25 genes of *Corcyra cephalonica*: molecular cloning, characterization, tissue-specific expression, synchronous developmental and 20-hydroxyecdysone regulation during the last instar larval development. Gen. Comp. Endocr. 167, 113–121. 10.1016/j.ygcen.2010.02.007 20171223

[B3] Chen F. Hesselberg T. Porter D. Vollrath F. (2013). The impact behaviour of silk cocoons. J. Exp. Biol. 216, 2648–2657. 10.1242/jeb.082545 23804671

[B4] Chen F. Porter D. Vollrath F. (2012a). Silk cocoon (*Bombyx mori*): Multi-layer structure and mechanical properties. Acta Biomater. 8, 2620–2627. 10.1016/j.actbio.2012.03.043 22484695

[B5] Chen F. Porter D. Vollrath F. (2012b). Morphology and structure of silkworm cocoons. Mat. Sci. Eng. C-Mater. 32, 772–778. 10.1016/j.msec.2012.01.023

[B6] Fahrbach S. E. Smagghe G. Velarde R. A. (2012). Insect nuclear receptors. Annu. Rev. Entomol. 57, 83–106. 10.1146/annurev-ento-120710-100607 22017307

[B7] Giebultowicz J. M. Zdarek J. U. Chróścikowska (1980). Cocoon spinning behaviour in *Ephestia kuehniella*: Correlation with endocrine events. J. Insect. Physiol. 26, 0–464. 10.1016/0022-1910(80)90116-X

[B8] Gilbert L. (2008). *Drosophila* is an inclusive model for human diseases, growth and development. Mol. Cell. Endocrinol. 293, 25–31. 10.1016/j.mce.2008.02.009 18374475

[B9] Guittard E. Blais C. Maria A. Parvy J. -P. Pasricha S. Lumb C.. (2011). CYP18A1, a key enzyme of Drosophila steroid hormone inactivation, is essential for metamorphosis. Dev. Biol. 349, 35–45. 10.1016/j.ydbio.2010.09.023 20932968

[B10] Guo X. Dong Z. Zhang Y. Li Y. Zhao P. (2016). Proteins in the cocoon of silkworm inhibit the growth of *Beauveria bassiana*. PLoS ONE 11:e0151764. 10.1371/journal.pone.0151764 27032085 PMC4816445

[B11] Iga M. Smagghe G. (2010). Identification and expression profile of Halloween genes involved in ecdysteroid biosynthesis in *Spodoptera littoralis*. Peptides 31, 456–467. 10.1016/j.peptides.2009.08.002 19682519

[B12] Kiyosawa M. Shirai I. E. Kanekatsu R. Miura M. Kiguchi K. (1999). Cocoon spinning behavior in the silkworm, *Bombyx mori*: comparison of three strains constructing different cocoons in shape. Zool. Sci. 16, 215–223. 10.2108/zsj.16.215

[B13] Li S. Yu X. Q. Feng Q. L. (2019). Fat body biology in the last decade. Annu. Rev. Entomol. 64, 315–333. 10.1146/annurev-ento-011118-112007 30312553

[B14] Li Z. Ge X. Ling L. Zeng B. Xu J. Asla A. F. You L. Palli S. R. Huang Y. P. Tan A. J. (2014). Cyp18a1 regulates tissue-specific steroid hormone inactivation in *Bombyx mori*. Insect Biochem. Molec. Biol. 54, 33–41. 10.1016/j.ibmb.2014.08.007 25173591 PMC4692384

[B15] Lin X. Y. Schutter K. D. Chafino S. Franch-Marro X. Martin D. Smagghe G. (2019). Target of rapamycin (TOR) determines appendage size during pupa formation of the red flour beetle *Tribolium castaneum*. J. Insect Physiol. 117, 0022–1910. 10.1016/j.jinsphys.2019.103902 31233769

[B16] Lounibos L. P. (1975). The cocoon spinning behaviour of the Chinese oak silkworm, *Antheraea pernyi*. Anim. Behav. 23, 843–853. 10.1016/0003-3472(75)90109-8

[B17] Lounibos L. P. (1976). Initiation and maintenance of cocoon spinning behaviour by saturniid silkworms. Physiol. Entomol. 1, 195–206. 10.1111/j.1365-3032.1976.tb00961.x

[B18] Miao Y. G. Shi L. G. Nair K. S. (2004). Ecdysteroid as a mediator in the regulation of silk protein synthesis and its influence on silkworm (Lepidoptera) genome. J. Appl. Entomol. 128, 348–353. 10.1111/j.1439-0418.2004.00854.x

[B19] Namiki T. Niwa R. Sakudoh T. Shirai K. Takeuchi H. Kataoka H. (2005). Cytochrome 450 CYP307A1/Spook: A regulator for ecdysone synthesis in insects. Biochem. Bioph. Res. Co. 337, 367–374. 10.1016/j.bbrc.2005.09.043 16188237

[B20] Niwa Y. Niwa R. S. (2014). Neural control of steroid hormone biosynthesis during development in the fruit fly *Drosophila melanogaster*. Genes Genet. Syst. 89, 27–34. 10.1266/ggs.89.27 24817759

[B21] Niwa Y. S. Niwa R. (2016). Transcriptional regulation of insect steroid hormone biosynthesis and its role in controlling timing of molting and metamorphosis. Dev. Growth Differ. 58, 94–105. 10.1111/dgd.12248 26667894 PMC11520982

[B22] Offord C. Vollrath F. Holland C. (2016). Environmental effects on the construction and physical properties of *Bombyx mori* cocoons. J. Mate. Sci. 51, 10863–10872. 10.1007/s10853-016-0298-5

[B23] Peng L. Wang L. Zou M. M. (2019). Identification of halloween genes and RNA interference-mediated functional characterization of a Halloween gene Shadow in *Plutella xylostella*. Front. Physiol. 10:1120. 10.3389/fphys.2019.01120 31555150 PMC6724230

[B24] Petryk A. Warren J. T. Marques G. Jarcho M. P. Gilbert L. I. Kahler J.. (2003). Shade is the *Drosophila* P450 enzyme that mediates the hydroxylation of ecdysone to the steroid insect molting hormone 20-hydroxyecdysone. Proc. Natl. Acad. Sci. U.S.A. 100, 13773–13778. 10.1073/pnas.2336088100 14610274 PMC283497

[B25] Pfaffl M. W. (2001). A new mathematical model for relative quantification in real-time RT–PCR. Nucleic Acids Res. 29:e45. 10.1093/nar/29.9.e45 11328886 PMC55695

[B26] Scieuzo C. Nardiello M. Salvia R. (2018). Ecdysteroidogenesis and development in, *Heliothis virescens*, (Lepidoptera: Noctuidae): focus on PTTH-stimulated pathways. J. Insect Physiol. 107, 57–67. 10.1016/j.jinsphys.2018.02.008 29454612

[B27] Sehnal F. Zurovec M. (2004). Construction of silk fiber core in Lepidoptera. Biomacromolecules 5, 666–674. 10.1021/bm0344046 15132645

[B28] Shi Y. Jiang H. B. Gui S. H. Liu X. Q. Pei Y. X. Li X.. (2017). Ecdysis triggering hormone signaling (ETH/ETHR-a) is required for the larva-larva ecdysis in *Bactrocera dorsalis* (Diptera: Tephritidae). Front. Physiol. 8:587. 10.3389/fphys.2017.00587 28878684 PMC5572281

[B29] Shimizu K. Ogawa S. Hino R. Adachi T. Tomita M. Yoshizato K. (2007). Structure and function of 5'-flanking regions of *Bombyx mori* fibroin heavy chain gene: Identification of a novel transcription enhancing element with a homeodomain protein-binding motif. Insect Biochem. Mole. Biol. 37, 713–725. 10.1016/j.ibmb.2007.03.016 17550827

[B30] Smagghe G. Degheele D. (1994). Effects of the ecdysteroid agonists RH 5849 and RH 5992, alone and in combination with a juvenile hormone analogue, pyriproxyfen, on larvae of *Spodoptera exigua*. Entomol. Exp. Appl. 72,115–123. 10.1111/j.1570-7458.1994.tb01809.x

[B31] Smagghe G. Gomez L. E. Dhadialla T. S. (2013). The bisacylhydrazine insecticides for selective pest control. Adv. Insect Physiol. 43,163–249. 10.1016/B978-0-12-391500-9.00002-4

[B32] Stuart A. E. Hunter F. F. (1995). A re-description of the cocoon-spinning behaviour of *Simulium vittatum* (Diptera: Simuliidae). Ital. J. Zool. 7, 363–377. 10.1080/08927014.1995.9522944

[B33] Su H. Cheng Y. Wang Z. Li Z. Stanley D. Yang Y. (2015). Silk gland gene expression during larval-pupal transition in the cotton leaf roller *Sylepta derogata* (Lepidoptera: Pyralidae). PLoS ONE 10:e0136868. 10.1371/journal.pone.0136868 26352931 PMC4564283

[B34] Sun M. J. Liu Y. Walker W. B. Liu C. C. Lin K. J. Gu S. H. Zhang Y. J. Zhou J. J. Wang G. R. (2013). Identification and characterization of pheromone receptors and interplay between receptors and pheromone binding proteins in the diamondback moth, *Plutella xylostella*. PLoS ONE 8:e62098. 10.1371/journal.pone.0062098 23626773 PMC3633919

[B35] Sutherland T. D. Young J. H. Weisman S. Hayashi C. Y. Merritt D. J. (2010). Insect silk: one name, many materials. Annu. Rev. Entomol. 55, 171–188. 10.1146/annurev-ento-112408-085401 19728833

[B36] Tang W. Q. Yu L. Y. He W. Y. Yang G. Ke F. S. Baxter S. W.. (2014). DBM-DB: the diamondback moth genome database. Database 2014:bat087. 10.1093/database/bat087 24434032 PMC3893660

[B37] Van der Kloot W. G. Williams C. M. (1953). Cocoon construction by the *Cecropia* silkworm. I. the role of the external environment. Behaviour 5, 141–156. 10.1163/156853953X00087

[B38] Xu J. Dong Q. L. Yu Y. Niu B. L. Ji D. F. Li M. W. Huang Y. P. Chen X. Tan A. J. (2018). Mass spider silk production through targeted gene replacement in *Bombyx mori*. Proc. Natl. Acad. Sci. U.S.A. 115, 8757–8762. 10.1073/pnas.1806805115 30082397 PMC6126722

[B39] Yagi N. (1926). The cocooning behavior of a *saturnian caterpillar* (*Dictyoploca japonica*); a problem in analysis of insect conduct. J. Exp. Zool. 46, 245–259. 10.1002/jez.1400460205

[B40] Yokoyama T. (1951). Studies on the cocoon-formation of the silkworm, *Bombyx mori*. Bull. Seri. Exp. Stat. 13, 183–260.

