# Supplementary figures and images for: Cocoon-Spinning Behavior and 20-Hydroxyecdysone Regulation of Fibroin Genes in Plutella xylostella
Source: Front Physiol. 2020 Dec 15;11:574800. doi: 10.3389/fphys.2020.574800 (PMC7770130; doi:10.3389/fphys.2020.574800)

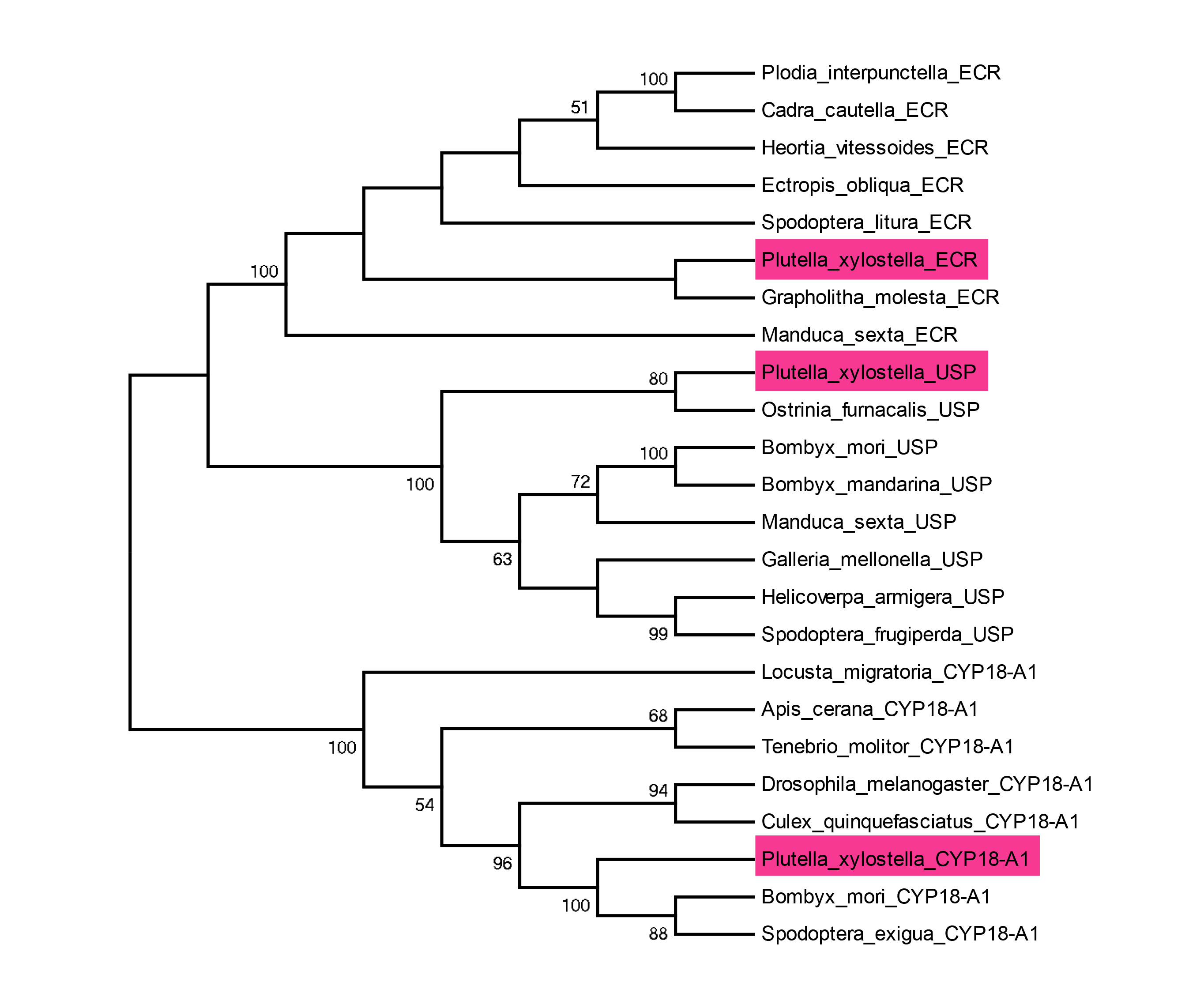

Supplement: Supplementary Figure 1 — Phylogenetic analysis of ECR, USP, and CYP18-A1 homologs from different insect species based on amino acid sequences. [file Image_1.TIF]
