# Supplementary material for: Cocoon-Spinning Behavior and 20-Hydroxyecdysone Regulation of Fibroin Genes in Plutella xylostella
Source: Front Physiol. 2020 Dec 15;11:574800. doi: 10.3389/fphys.2020.574800 (PMC7770130; doi:10.3389/fphys.2020.574800)
Supplement: Supplementary Table 1 — Primer sequences used in this study. [file Table_1.DOC]

**Table S1. Primer sequences used in this study**

| Target | Direction | Sequence 5’ to 3’ | Tm value PCR product size Access No. |  |
| --- | --- | --- | --- | --- |
| *Fib-H* (Cloning) | Forward | ATGAGAGCAGCAACCTTTGT | 55℃ 318bp MN935438 |  |
|  | Reverse | TTAGCAATTGACACAAGGTG |  |  |
| *Fib-L* (Cloning) | Forward | ATGCTGCCTATAGTGTTGG | 56℃ 759bp MN935439 |  |
|  | Reverse | CCATCCGCTCCGCCGTCTAG |  |  |
| *P25* (Cloning) | Forward | ATGATCTATTTTTATCTGCTGG | 65℃ 645bp MN935437 |  |
| *EcR* (Cloning)  *USP* (Cloning)  *CYP18-A1* (Cloning) | Reverse  Forward  Reverse  Forward  Reverse  Forward  Reverse | TTAGTAAATTTTACAACCAA  ATGAAACGCCACTGGTCCAAC  CTAGAGCTCGGGGGCGTCGA  ATGGAGCCCGGAAGAGAAGC  CTACATCATAGAGTTAGTATC  ATGTTTTCGAACTCGAAGCT  CTAGTGGCAGCCGACGTTGC | 57℃ 1638bp MT629927  63℃ 1245bp MT629928  59℃ 1596bp MT629929 |  |
| *Fib-H* (qRT-PCR) | Forward | AATGGAACCGTCTTTGAACG | 60℃ 102bp |  |
|  | Reverse | CAACTTTTTCGTCGCTGTGA |  |  |
| *Fib-L* (qRT-PCR) | Forward | CGACGGATGCGAATACACTA | 60℃ 113bp |  |
|  | Reverse | TTGACGATGGTGTCGATGTT |  |  |
| P25 (qRT-PCR) | Forward | TTCGAAACCCCCTACTTCAA | 60℃ 126bp |  |
|  | Reverse | ATCACAGCCTTATCCGTTGC |  |  |
| *rpl32* (internal reference) | Forward | CCAATTTACCGCCCTACC | 60℃ 135bp |  |
|  | Reverse | TACCCTGTTGTCAATACCTCT |  |  |
| *Actin* (internal reference) | Forward | GCCGTCTTCCCGTCCAT | 60℃ 117bp |  |
|  | Reverse | GATACCTCTCTTGCTCTGGGC |  |  |
